# Food Label Literacy and Use among US Adults Diagnosed with Cancer: Results from a National Representative Study

**DOI:** 10.1007/s13187-018-1403-z

**Published:** 2018-07-26

**Authors:** Ann Oyare Amuta-Jimenez, Celia Lo, Divya Talwar, Nicole Khan, Adam E. Barry

**Affiliations:** 1 Department of Health Studies, Texas Woman’s University, Denton, TX USA; 2 Department of Sociology, Texas Woman’s University, Denton, TX USA; 3 Texas A&M University, College Station, TX USA; 4 Texas Woman’s University, Denton, TX 77843 USA; 5 Department of Health and Kinesiology, Texas A&M University, Mail Stop 4243, College Station, TX 77843 USA

**Keywords:** Cancer control, Nutrition, Food label use, Food label literacy

## Abstract

For those diagnosed with cancer, lifestyle factors including diet can be more important than ever. However, lack of nutrition-related knowledge can pose a significant barrier to healthy eating. Food labels guide consumers in selecting appropriate portion sizes—that is, caloric content—and ensuring adequate intake of nutrients. Data from the 2013-2014 HINTS were used to examine (a) differences in food label use and food label literacy between respondents ever had a cancer diagnosis and those never had a diagnosis; (b) sociodemographic correlates and health-related correlates of food label use and literacy, in a context of cancer diagnosis; and (c) potential association between food label use/literacy and each of two dietary choices, eating vegetables and fruits and limiting intake of sugary drinks, again, in a context of cancer diagnosis. Data was analyzed via SPSS version 24.0, and cross tabulations using Pearson's Chi-square test and logistic regressions. Income, gender and non-participation in support groups were associated with food label literacy (p<.05). Confidence to take care of self was associated with food label use (p<.05). Relationships were observed between using food labels and curtailing soda intake (b = -.368, p<.05), eating relatively more fruits (b = .558, p<.05), and eating relatively more vegetables (b = .558, p<.05). The overall models predicting consumption of soda [x2 (2) = 13.70, p = .001, Nagelkerke R-square = .059], of fruits [x2 (2) = 33.87, p < .001, Nagelkerke R-square = .136], and of vegetables [x2 (2) = 36.08, p < .001, Nagelkerke R-square = .144] was statistically significant. Implications for research and practice can be found in results linking food label use to better quality diets. They include the usefulness of nutrition education interventions targeting lower-income men with cancer diagnoses; one lesson should be the use of food labels.

## Introduction

Cancer is the second most common cause of mortality in the USA; approximately 1,735,350 new cases will be diagnosed in 2018 [46]. As technology progresses, diagnosis and treatment become more efficient [5]. Entering the twenty-first century, the number of cancer diagnoses in the USA has increased rather than fallen off, which has been attributed to [1] earlier diagnosis, [2] improved treatment, and [4] an aging population [16]. Currently, Americans diagnosed with all types of cancer have an average survival rate of 34% [5]. Between new diagnoses and rising rates of survival, each year finds millions of Americans experiencing a variety of cancer-related psychosocial and emotional challenges [39].

After completing treatment, cancer survivors do not just face psychological distress, they become relatively more susceptible to chronic illnesses including cardiovascular disease, type 2 diabetes, and obesity [17, 25, 39]. The latter is especially common in women who have received treatment for breast cancer. In this group, weight gain resulting from treatment averages 2.5–6.2 kg [42]. For those diagnosed with cancer, lifestyle factors including diet can be more important than ever. Diagnosis often motivates healthier lifestyle choices that can improve health outcomes and even reduce the likelihood of cancer recurrence [39, 42]. A study of postmenopausal women diagnosed with cancer found that food choices following diagnosis impacted patients’ mental and physical well-being and were linked to likelihood of relapse [25]. Despite potential benefits of a good diet after cancer, research has not always found prior diagnosis to be associated with healthy eating [31]. For some cancer survivors, according to a group of surveyed healthcare providers, lack of nutrition-related knowledge can pose a significant barrier to healthy eating [12].

The American Cancer Society (ACS) publishes a booklet, *Nutrition and Physical Activity Guidelines*, intended to familiarize cancer patients with the use of mandated food labels. Labels guide consumers in selecting appropriate portion sizes—that is, caloric content—and ensuring adequate intake of nutrients [27]. The booklet states that ACS recommends a plant-based diet, prescribing daily consumption of at least 2.5 cups of vegetables and fruits, along with whole grains, and advising limited consumption of processed foods and red meat [3, 27, 45].

Since passage of the National Labeling and Education Act in 1990, all marketers of packaged food products have had to disclose products’ calorie and nutrient content and other information, using a prescribed easy-to-read format known as “Standardized Nutrition Facts” [44]. The act requires each food label to list nutrients present in the product and indicate the proportion of daily nutritional requirements each serving of the product represents. Each label informs consumers about, especially, the fats and cholesterol present in the product, along with the sodium, sugar, protein, and certain vitamins, so that healthy foods are distinguished from less healthy ones. Using food labels to ensure a nutritious, healthy diet is considered a preventive measure against cancer [37].

But a particular literacy is required to use food labels well. Reading labels without genuinely comprehending the information they present may lead to disappointing results. *Food label literacy*, a subset *of health literacy*, means the ability to obtain, process, and understand nutrition information from food labels leading to healthful food-related decisions such as those on calorie intake [50]. Food label literacy would seem imperative in the wake of a cancer diagnosis. However, little research to date has explored nutritional knowledge and food label use and literacy among chronically ill individuals, specifically those having cancer [19].

The present study used the 2013–2014 Health Information National Trends Survey (HINTS) to examine (*a*) differences in food label use and food label literacy between respondents who have had a cancer diagnosis and those who have never had a diagnosis; (*b*) sociodemographic correlates and health-related correlates of food label use and literacy, in a context of cancer diagnosis; and (*c*) potential association between food label use/literacy and each of two dietary choices, eating vegetables and fruits and limiting intake of sugary drinks, again, in a context of cancer diagnosis. The study offers three contributions to the literature. First, to our knowledge, it is the first research to focus on the food label use and literacy of, specifically, Americans diagnosed with cancer. Second, it features relatively vigorous response rates, due to the use of a nationally representative dataset HINTS obtained by employing multiple sampling methods. Third, and importantly, it promotes oncological health. Most cancer survivors are advised to make certain lifestyle changes to ward off cancer recurrence and other chronic disorders to which cancer makes them susceptible; lifestyle changes can also boost physical and mental health. Perhaps paramount is changing one’s diet (frequently accompanied by increasing one’s physical activity). Excess body weight is a cancer risk factor. Moreover, cancer treatment often alters the individual’s capacity for food digestion and nutrient absorption, and dietary assessment is a key component of rehabilitation [5]. Appropriate use of food labels helpfully informs such individuals as to the amount of needed nutrients a food product provides, and at what caloric cost.

## Methods

### Data and Sample

We used the 2013–2014 HINTS to generate our study data [34]. A biennial national survey monitoring change in the public’s medical environment over time, HINTS is a mail survey targeting non-institutionalized individuals 18 years or older and providing new data both for the National Cancer Institute’s outreach effort and its cancer research effort [36]. For the HINTS dataset we accessed, the research strategy had involved oversampling Hispanic/Latino and Black/African Americans during data collection September–December 2013. HINTS researchers claimed an overall response rate of 35.2% for this survey; a total of 3185 mailed-in questionnaires deemed to meet study criteria were included in the research. The present data analysis employed appropriate sampling weights obtained from HINTS [1, 2]. Further information about HINTS is available elsewhere [18, 36]. The institutional review board of the university with which the authors are affiliated exempted the present secondary investigation from review.

### Measures

**Fig. 1:**
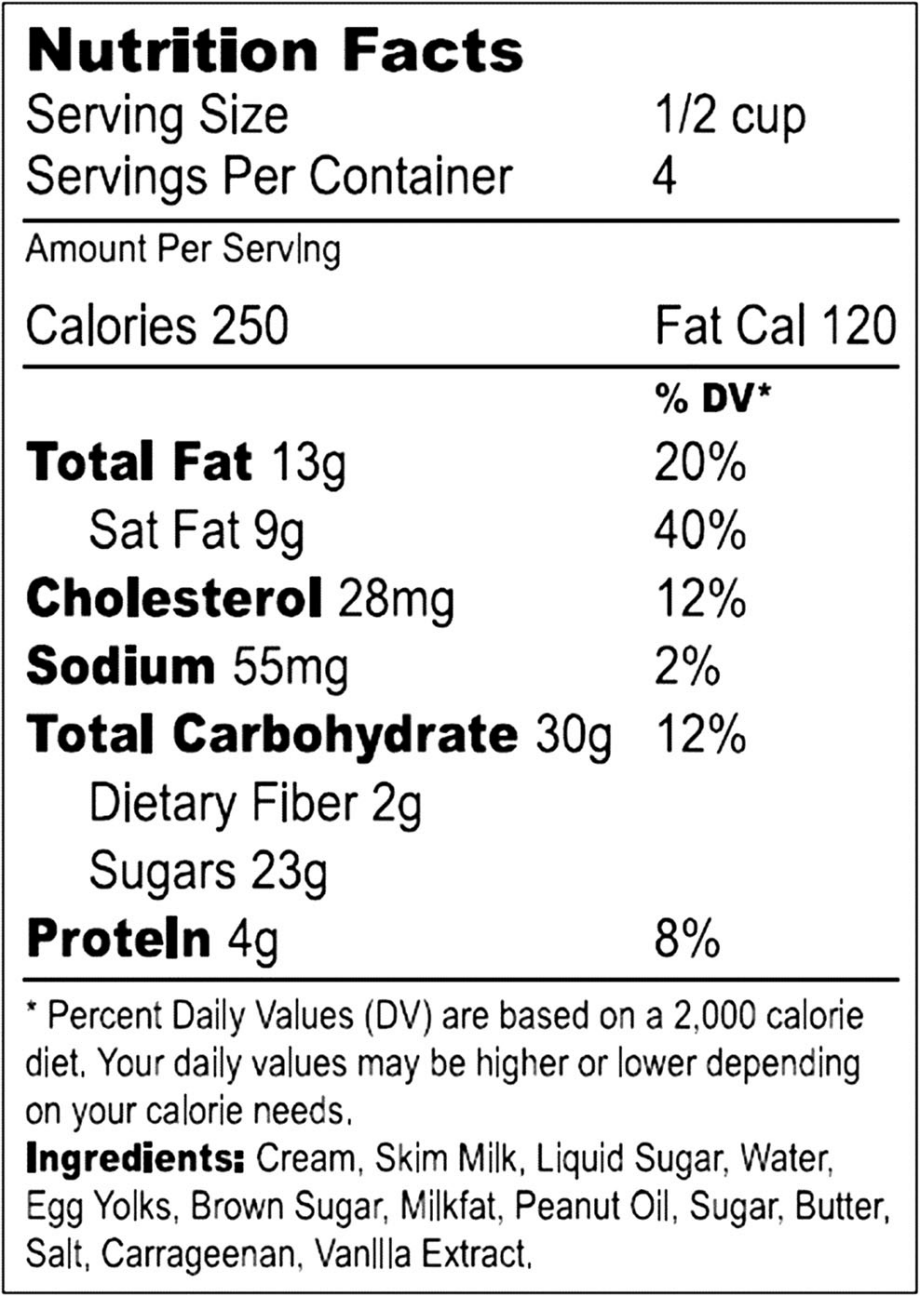
Showing sample food label shown to participants. Answers that measured food label literacy were based on the questions answered from this example

We posed three research questions, employing as outcome variables *food label use*, *food label literacy*, and *dietary behaviors*. In HINTS, participant label use was assessed by asking, “When available, how often do you use menu information on calories in deciding what to order”? Offered responses were “Never” (coded as *0*), “Rarely” [1] “Sometimes” [3] “Often” [4] and “Always” [7]. To measure food label literacy, the questionnaire first presented respondents with a sample label (see Fig. 1) from an ice cream product [1, 2]. A prompt below this product’s label read, “The food label above can be found on the back of a container of a pint of ice cream. We would like to know how easy it is to use this information. Use the food label above to answer questions.” There were four questions, as follows: “If you eat the entire container, how many calories will you eat?”; “If you are allowed to eat 60g of carbohydrates as a snack, how much ice cream could you have?”; “Your doctor advises you to reduce the amount of saturated fat each day, which includes 1 serving of ice cream. If you stop eating ice cream, how many grams of saturated fat would you be consuming each day?”; and “If you usually eat 2,500 calories in a day, what percentage of your daily value of calories will you be eating if you eat one serving?” Correct answers for these questions were, respectively, 1000 cal, 1 cup, 33 g, and 10%. Respondents answering each question correctly were assigned a *1* for that question. We used measures of the dichotomous variables (derived from the survey item about label literacy) to create one composite variable measuring food label literacy on a scale of 0 to 4; higher values indicated greater literacy.

Consumption of soda, of fruits, and of vegetables were self-reported and allowed to indicate dietary behaviors. To measure consumption of soda, participants were asked to indicate this behavior’s frequency on a 1–6 Likert scale, with 6 indicating daily consumption and 1 indicating occasional consumption. To measure consumption of fruits, participants were asked how many cups of fruits they consumed, on average, each day, with offered responses ranging from 0 (*none*) to 6 (*4 cups or more*). To measure consumption of vegetables, they were asked how many cups of vegetables they consumed each day, with offered responses again ranging from 0 (*none*) to 6 (*4 cups or more*).

Study data also included several indicators of general health: body mass index (*BMI*), *confidence in ability to take care of self*, *health information–seeking behavior*, and *participation in cancer support group*. The variables were pertinent, having been found to influence health-related behavior (not specifically among Americans diagnosed with cancer, however, but among a general population. *Confidence in ability to take care of self* was measured via a Likert scale ranging from 1 (*not confident at all*) to 5 (*completely confident*) [6, 7, 24, 26, 47]. *BMI* was a continuous measure derived by comparing weight to height in inches. *Health information–seeking behavior* was measured dichotomously based on whether respondent had ever sought information on cancer from any source [1] or never sought it (0). Finally, *participation in cancer support group* was measured dichotomously (1 = yes, 0 = no) based on whether, in the year preceding survey, respondent had participated in an online forum or other group for people similarly diagnosed.

Personal history of cancer was assessed dichotomously (1 = yes, 0 = no) by asking respondents, “Have you ever been diagnosed as having cancer”? In addition, since in the general population, food label use and literacy appear to be explained to some degree by demographic and social status variables (e.g., gender, race/ethnicity, age, income, marital status, education, English literacy), we included such variables in our models [29, 32, 38, 41].

### Statistical Procedures

To compare cancer patients’ food label use and food label literacy to other people’s, we used SPSS (version 24) to conduct, respectively, Chi-square testing and *t*-testing. A total of 3135 respondents were included in these bivariate analyses. Next, we used ordinal logistic regression—and a sample (*n* = 459) limited to cancer-diagnosed respondents—to evaluate (*a*) whether and how health-related variables explain food label use and food label literacy, controlling for sociodemographic variables and (*b*) whether and how food label use and food label literacy explain dietary behaviors. Each health factor variable was added blockwise, producing five different blocks. By including the health factor variables blockwise in our model, it becomes apparent what the individual, distinct contribution of each health factor variable is in further understanding food label use and literacy.

## Results

Table 1 shows descriptive statistics for all included variables. The sample of cancer-diagnosed respondents was primarily female (*n =* 280; 61% women) and the majority had at least some college education (*n =* 252; 54.6%), were married (*n =* 225; 50.4%), were White (*n =* 285; 76.2%), and reported annual income under USD 50,000 (*n =* 229; 52.1%). Respondents’ average age was 66.1 years (SD = 13.7) and average BMI was 28.02 (SD = 6.18). An average of 28% respondents consumed 1–2 cups of vegetables and 1–2 cups of fruits per day. Most respondents (49.5%) did not drink any soda or pop, majority (93%) did not participate in a cancer support group, majority (49%) felt confident that they could care for themselves, and most (81%) search for health information. Several respondents (38.3%) had four correct answers to the food literacy questions. Several respondents (29.5%) used food labels “sometimes.” Our evaluation of potential relationship between cancer diagnosis and food label use, and between diagnosis and food label literacy, yielded no statistically significant differences distinguishing cancer-diagnosed respondents from other respondents in terms of their use of [*x*^2^ [7] = 5.45, *p* = .244, Cramer’s *V* = .042] and fluency with [*t* (1,731) = .13, *p* = .899] food label information.

**Table 1:** Descriptive statistics for demographics, health status, and dietary variables

Variables	Number		Percentage	
Gender				
Male	168		37.5	
Female	280		62.5	
Race/Ethnicity				
Hispanic	37		9.9	
Caucasian	285		76.2	
African American	36		9.6	
Asian	7		1.9	
Other	9		2.4	
Marital status				
Married	225		50.4	
Single	39		8.7	
Divorced	81		18.2	
Other	101		22.6	
Income				
$0 to $9999	33		7.5	
$10,000 to $14,999	40		9.1	
$15,000 to $19,999	36		8.2	
$20,000 to $34,999	51		11.6	
$35,000 to $49,999	69		15.7	
$50,000 to $74,999	86		19.5	
$75,000 to $99,999	54		12.3	
$100,000 to $199,999	47		10.7	
$200,000 or more	24		5.5	
Level of education				
Less than high school degree	46		10.3	
High school degree	106		23.8	
Some college	132		29.6	
College graduate	95		21.3	
Postgraduate	67		15.0	
Seeks health information				
No	87		19	
Yes	372		81	
Perceived ability to taking care of self				
Not confident at all	11		2.5	
A little confident	12		2.7	
Somewhat confident	119		27	
Very confident	217		49.2	
Completely confident	82		18.6	
Participates in a support group				
No	279		93.3	
Yes	20		6.7	
Soda consumption				
I don’t drink any regular soda or pop	225		49.5	
Less often than 1 day a week	103		22.6	
1 to 2 days a week	58		12.7	
3 to 4 days a week	29		6.4	
5 to 6 days a week	10		2.2	
Every day	30		6.6	
Fruit consumption				
None	41		9.2	
1/2 cup or less	74		16.6	
1/2 cup to 1 cup	125		28.1	
1 to 2 cups	125		28.1	
2 to 3 cups	44		9.9	
3 to 4 cups	29		6.5	
4 or more cups	7		1.6	
Vegetable consumption				
None	23		5.1	
1/2 cup or less	53		11.9	
1/2 cup to 1 cup	116		26	
1 to 2 cups	126		28.2	
2 to 3 cups	78		17.4	
3 to 4 cups	35		7.8	
4 or more cups	16		3.6	
Age				
	Min		24	
	Max		96	
	M		66.12	
	SD		13.73	
BMI				
	Min		10.6	
	Max		55.7	
	M		28.02	
	SD		6.18	
Food label use				
Never	102		22.2	
Rarely	104		22.9	
Sometimes	134		29.5	
Often	76		16.7	
Always	39		8.6	
Food label literacy				
0 correct answers	13		5.3	
1 correct answer	17		7.0	
2 correct answers	56		23.0	
3 correct answers	64		26.3	
4 correct answers	93		38.3	

Table 2 shows ordinal logistic regression results for food label use predicted by our sociodemographic variables and by our general-health indicators, *BMI*, *confidence in ability to take care of self*, *health information–seeking behavior*, and *participation in cancer support group*. No general-health indicator proved statistically significant in this model. Analysis did link use of food labels to higher income (*b* = .166, *p* < .05) and female gender (*b* = −.682, *p* < .05). The overall model was found to improve explanation of food label use [*p* < .05; Nagelkerke R-square = .103].

**Table 2:** Ordinal logistic regression predicting food label use

	Model 1 ***	Model 2 ***	Model 3 ***	Model 4 ***	Model 5 **
Age	−0.021 *	−0.021 *	−0.021 *	−0.021 *	−0.006
Education	0.205	0.193 *	00.2 *	0.168	0.175
Income	0.054	0.055	0.062	0.045	0.166 *
Speak English	−0.087	−0.134	−0.16	−0.162	−0.628
Occupation status	0.018	0.04	0.027	0.014	−0.033
Married	1.061	0.923	0.996	0.922	1.049
Single	0.651	0.458	0.463	0.381	0.718
Divorced	1.341	1.146	1.211	1.068	1.516
Other marital status	0.882	0.673	0.655	0.521	0.761
Gender	−0.613 *	−0.631 *	−0.611 *	−0.537 *	−0.682 *
White	−0.191	−0.121	−0.175	−0.168	0.305
Black	−0.471	−0.368	−0.394	−0.474	−0.058
Asian	−0.154	−0.015	−0.123	−0.282	0.305
Hispanic	−0.316	−.166	−.150	−0.208	−0.680
Other race	0.476	0.448	0.419	0.514	1.255
Ability to take care of self	–	0.259 *	0.263 *	0.258 *	0.185
Body mass index	–	–	0.013	0.012	0.002
Seeks health information	–	–	–	−0.759 *	−0.549
Participates in a support group	–	–	–	–	−0.786 *
Nagelkerke R-square	0.103	0.115	0.124	0.145	0.163
Change in Nagelkerke R-Square	–	0.012	0.009	0.021	0.018

*Significant at .05 level, **significant at .01 level, ***significant at .001 level

Table 3 presents observed associations between food label literacy and our sociodemographic variables and general-health indicators. Analyses with the ordinal logistic regression technique suggested that, among cancer-diagnosed respondents, greater food label literacy was linked to high income (*b* = .267, p < .05), low BMI (*b* = −.65, *p* < .05), and nonparticipation in support group (*b* = −.591, *p* < .05). The overall model proved to be statistically significant: *x*^2^ [26] = 46.21, *p* < .001, and Nagelkerke R-square = .156.

**Table 3:** Ordinal logistic regression predicting food label literacy

Predictor variables	Block 1 ***	Block 2 ***	Block 3 ***	Block 4 ***	Block 5
Age	−0.018	−0.018	−0.019	−0.019	−0.005
Education	0.05	0.015	0.015	−0.006	−0.059
Income	0.317 ***	0.329 *	0.336 ***	0.324 ***	0.267 **
Speak English	−0.042	−0.142	−0.092	−0.041	0.258
Occupation status	0.007	0.026	0.034	0.023	0.005
Married	−0.765	−0.936	−1.048	−1.089	−1.161
Single	−1.128	−1.162	−1.155	−1.235	−1.538
Divorced	−1.287	−1.503	−1.608	−1.699	−1.281
Other marital status	−0.889	−0.994	−1.169	−1.232	−1.113
Gender	−0.071	−0.103	−0.077	−0.04	0.084
White	−0.751	−0.897 *	−0.889 *	−0.858	0.141
Black	0.948	0.829	0.607	0.563	1.17
Asian	−0.273	−0.297	−0.161	−0.212	−0.642
Hispanic	−0.508	−0.800	−0.720	−0.859	−0.751
Other race	−0.941	−1.026	−1.279	−1.221	0.246
Confidence to take care of self	–	0.216	0.192	0.177	−0.047
Body mass index	–	–	−0.50 *	−0.51 *	−0.65 *
Seek health information	–	–	–	−0.401	−0.284
Participates in a support group	–	–	–	–	−0.591*
Nagelkerke R-square	0.208	0.221	0.239	0.244	0.156
Change in Nagelkerke R-Square	–	0.013	0.018	0.005	−0.088

*Significant at .05 level, **significant at .01 level, ***significant at .001 level

### Dietary Behaviors and Food Label Literacy and Use

Table 4 presents ordinal regression results predicting the three outcome dietary behaviors, consumption of soda, of fruits, and of vegetables. Across all models, food label use was the sole factor observed to be linked to healthy dietary behaviors. Relationships were observed between using food labels and curtailing soda intake (*b* = −.368, *p* < .05), eating relatively more fruits (*b* = .558, *p* < .05), and eating relatively more vegetables (*b* = .558, *p* < .05). The overall models predicting consumption of soda [*x*^2^ (2) = 13.70, *p* = .001, Nagelkerke R-square = .059], of fruits [*x*^2^ (2) = 33.87, *p* < .001, Nagelkerke R-square = .136], and of vegetables [*x*^2^ (2) = 36.08, *p* < .001, Nagelkerke R-square = .144] was statistically significant.

**Table 4:** Ordinal regressions predicting soda, fruit, and vegetable consumption

Predictor variables	Soda consumption	Fruit consumption	Vegetable consumption
Food label literacy	−0.068	0.135	0.189
Food label use	−0.368 *	0.558 **	0.558
Model *p*-value	0.001	< .001	<.001
Nagelkerke R-square	0.059	0.136	0.144

*Significant at .05 level, **significant at .01 level, ***significant at .001 level

## Discussion

This study was prompted by the numerous negative consequences reported to accompany weight gain, obesity, and associated chronic illness in people diagnosed at some point with cancer [17, 25, 39]. Cancer patients’ and survivors’ enhanced susceptibility to the three prompted the American Cancer Society to publish *Nutrition and Physical Activity Guidelines*, which encourages this group’s food label literacy and use of food labels [27]. We sought evidence on how cancer diagnosis might be related to use of food labels and food label literacy; evidence on how patients’ / survivors’ sociodemographic variables and general-health indicators might be linked to their food label use and literacy; and evidence on how food label use and literacy might in turn help explain dietary behaviors of cancer-diagnosed respondents.

Central to our inquiry, we observed no differences between the food label use or food label literacy of respondents diagnosed with cancer and respondents not diagnosed with cancer. Ordinal logistic regression showed few of the selected sociodemographic variables and general-health indicators we tested to exhibit association with food label use or food label literacy. Moreover, while we found a link between food label use and dietary behaviors, we did not find one between food label literacy and dietary behaviors. These results foster four interpretations yielding reasonable implications.

First, per the results, higher-income females are more likely to use food labels, and food label literacy is most likely among higher-income people, those with relatively low BMI, and those participating in a support group. Earlier research similarly showed females generally to be more likely than males to use food labels (but it also suggested—as our findings do not—a role for gender in food label literacy) [11, 13, 35, 48]. Such results are unsurprising, since in so many households it is women, not men, who plan meals and shop for food. Women’s more extensive use of food labels is certainly understandable given this reality.

The significant positive relationships we found between income and food label use/literacy may be inconsistent with many previous research findings. For example, Pérez-Escamilla & Haldeman [40] worked with a national representative sample and reported no significant difference in food label use by people of relatively high socioeconomic status compared to those of low socioeconomic status. Additionally, prior studies have documented a link between education (a socioeconomic factor) and the accurate use of food labels [19, 37, 43, 44], but our results identified income alone as a socioeconomic factor associated with label use/literacy. In any case, because income dictates the amount and quality of foods that can be purchased, it is reasonable that our study observed income to be relevant in food label use/literacy. Still, using food labels does indeed imply cancer survivors’ behavioral change.

In our study, BMI and support group participation were significantly associated with food label literacy. Benefits of support groups include understanding, physical and emotional comfort, exchange with others experiencing one’s own illness, and encouragement. But our study found participation to lessen likelihood of food label literacy. The implication is that support groups may expose participants to incorrect or misleading information, along with the beneficial experiences. Rarely are support groups facilitated by health professionals, although that might discourage misinformation. Concerning BMI, which we found here to be associated with food label literacy in a negative direction, it may be that among cancer survivors, lower BMI and enhanced food label literacy coexist. With our merely cross-sectional dataset, we could not address causal relationship.

Second, and consistent with previous research [12, 20, 33, 43], we observed healthy dietary behaviors to increase significantly in the presence of food label use. (Not-significant results were found for food label literacy and may imply that using labels in and of itself demonstrates a sufficient level of food label literacy to prompt healthy dietary behaviors.) Those who used labels were likely to consume fewer sodas and more fruits and vegetables. Such results confirm older research indicating that when people used food labels, the diet they ate was relatively likely to include many fruits and vegetables and relatively few less-healthy foods.

Third, our finding of no significant difference between the food label use/literacy of cancer-diagnosed respondents and that of other respondents contrasts with previous findings. An example is the reportedly stronger nutritional knowledge and more-frequent use of food labels by those having chronic illness like cardiovascular disease or type 2 diabetes [11, 22, 29]. What we observed, however, would indeed be consistent with a recent report that 50–70% of cancer survivors do not adhere to dietary recommendations [49]. Our findings’ discrepancy with findings from investigations of chronic conditions besides cancer perhaps reflect the lack of consensus on nutrition’s role in cancer prevention, as opposed to the firm consensus on its very clear role in type 2 diabetes and cardiovascular disease [9, 30]. To benefit cancer patients and survivors, researchers need to explore and understand inconsistent implementation of survivorship care plans, focusing on healthcare providers’ role. Studies show the chronically ill are more likely to use food labels when instructed in basic nutrition science by healthcare providers (versus the chronically ill never consulting a provider about nutrition) [29, 41]. One study links physician recommendation to increased use of food labels [41]. Healthcare providers may prove to be effective agents fostering use of food labels by patients.

The transtheoretical model of behavior change, for which previous studies have obtained evidence, offers to explain breast and prostate cancer patients’ postdiagnosis healthy food choices [14, 15, 21, 45]. However, we should doubtless expect differences in preventive steps taken by cancer patients in an “action phase” versus cancer survivors in a “maintenance phase.” Moreover, the two groups may tend to perceive distinctly their relative susceptibility to cancer. That is, those coming to terms with diagnosis and treatment could feel defeated, asking what would be the point of reading food labels when they have this diagnosis; those who have completed treatment and heard they are cancer-free may feel motivated to practice healthier eating to prevent a recurrence of cancer.

Fourth, implications for research and practice can be found in our results linking food label use to better quality diets. They include the usefulness of nutrition education interventions targeting lower-income men with cancer diagnoses; one lesson should be the use of food labels. The best strategies for delivering nutrition education interventions for all demographics need to be identified by researchers. The literature does show that physicians’ and other healthcare providers’ involvement could be key in food label use by primary care and specialty care patients [29, 41]; these professionals should make much of the fact that preventing cancer relapse is possible through lifestyle change [8, 28]. Augmenting such interpersonal healthful nutrition advocacy among cancer patients and survivors should be well-designed national campaigns.

Future studies on our topic are warranted. There is a need to explore factors beyond demographic variables and general-health indicators that, too, might shape cancer patients’ and survivors’ use of food labels. Furthermore, research is needed on how healthcare providers currently discuss food choices with cancer patients and on the best strategies for ensuring use of food labels. We remain unaware of something as basic as timing: When exactly during the diagnosis–treatment course are patients most likely to begin using food labels? It would be useful to know. Lastly, it is worthy to note that food labels have been in constant change over the past few years and will continue to be as the FDA continues to explore ways to make understanding food labels and vitamin names easier, thus, continuous research is warranted.

Two limitations characterize our study and thus its results. First, our analyses were cross-sectional, precluding assertion of causal relationships, notably any between food label use and dietary behaviors. Second, we were constrained by the questions specific to the HINTS survey instrument. We measured food label literacy use and literacy using what was available in the HINTS dataset, despite its several discrepancies with prior food label use/literacy studies [23]. Our data’s secondary nature also meant we could not consider in our analyses any of the important barriers to healthy food choices, for instance proximity of full-service grocers. While we do not consider it a study limitation, it should also be noted that we looked on receipt of cancer diagnosis as a proxy for cancer survivor status. Survivor status should properly designate only individuals who were diagnosed with cancer but at the conclusion of treatment were deemed free of the disease [10]. Some in our sample might have been classified as cancer survivors while in fact they continued to fight the disease. In future research, it would be useful to specifically and correctly observe whether each respondent’s cancer was actually in remission.
